# The value of lymphocyte-to-C-reactive protein ratio for predicting clinical outcomes in patients with sepsis in intensive care unit: a retrospective single-center study

**DOI:** 10.3389/fmolb.2024.1429372

**Published:** 2024-09-13

**Authors:** Chao Song, Zhenkui Hu, Jinhui Zhang

**Affiliations:** ^1^ Department of Emergency Medicine, The Affiliated Hospital, Jiangsu University, Zhenjiang, Jiangsu, China; ^2^ Department of Critical Care Medicine, The Affiliated Hospital, Jiangsu University, Zhenjiang, Jiangsu, China

**Keywords:** sepsis, lymphocyte-to-C-reactive protein ratio, acute kidney injury, inflammation, immune

## Abstract

**Background:**

The lymphocyte-to-C-reactive protein ratio (LCR) was a novel biomarker of inflammation that had been implicated in various diseases. Nevertheless, the role of LCR in the context of sepsis patients admitted to the Intensive Care Unit (ICU) had not been thoroughly elucidated. This study aimed to determine the significance of the LCR in predicting the prognosis of sepsis patients within ICU.

**Methods:**

A sample of sepsis patients requiring ICU care was selected from the Affiliated Hospital of Jiangsu University. These patients were then segmented into four quartiles based on their LCR levels. The primary endpoint of the study was 30-day mortality and the secondary endpoint was the occurrence of Acute Kidney Injury (AKI). Survival analysis, via the Kaplan-Meier method and log-rank test, was conducted to assess survival rates. Cox proportional hazards regression and logistic regression models were employed to investigate the association between LCR and clinical outcomes. Additional subgroup analyses were conducted to evaluate the influence of other confounding factors on the relationship between LCR and patient outcomes.

**Results:**

A total of 1,123 patients were enrolled in this study, with a median age of 75 (65–84) years, and 707 (63.0%) of them were male. The 30-day mortality rate was 28.1%, while the incidence of AKI was 45.6%. A progressive decrease in LCR levels was found to be associated with an increased cumulative incidence of 30-day mortality (log-rank *P* < 0.001). Multivariable Cox proportional hazards analyses demonstrated that LCR was an independent predictor of 30-day mortality [per 1-unit increase in LCR: HR (95%CI): 0.370 (0.142–0.963); *P* = 0.042]. Additionally, multivariable logistic regression analysis revealed a significant association between LCR and AKI occurrence [per 1-unit increase in LCR: OR (95%CI): 0.541 (0.307–0.953); *P* = 0.034]. Furthermore, subgroup analysis indicated a stronger correlation for patients aged over 65 years compared to those aged 65 or younger (*p* for interaction <0.05) in predicting 30-day mortality or AKI occurrence based on LCR.

**Conclusion:**

A reduction in LCR was notably linked to 30-day mortality and the occurrence of AKI in sepsis patients. These findings suggested that LCR could potentially serve as a valuable tool in identifying sepsis patients at a heightened risk of adverse outcomes.

## Introduction

Sepsis is a condition that can be fatal due to an malfunctioning immune response to an infection. Currently, there are no approved specific treatments for sepsis (Chen and Wei, 2021). In developed countries, the rate of sepsis cases per 100,000 individuals had been estimated to be 437 over the past decade, with an adult mortality rate of about 17 percent. Severe sepsis, which had a higher severity, had an annual incidence of 270 cases per 100,000 people and a mortality rate of 26%. Developing and less developed countries tend to have even higher incidence and mortality rates of sepsis (Fleischmann et al., 2016). Due to the serious nature of sepsis and its associated poor prognosis, researchers had focused on identifying various risk factors that can predict the outcome of patients with critical illness (Jones et al., 2009; Gilani et al., 2014). Nevertheless, despite these endeavors, the fatality rate linked to sepsis persisted at a high level. It was therefore essential to promptly recognize and forecast the likelihood of death in individuals with sepsis to assess the gravity of the condition and determine suitable treatment courses.

The development of sepsis was associated with an imbalance between pro-inflammatory and anti-inflammatory responses (Singer et al., 2016; Chousterman et al., 2017; Liu and Sun, 2019). During the early stages of sepsis, activated immune cells released large quantities of pro-inflammatory factors, leading to a hyperimmune response and the onset of a cytokine storm (Hahn et al., 2016). C-reactive protein (CRP), synthesized in the liver, served a critical function as an inflammatory agent combating bacterial infection and sepsis (Black et al., 2004). Since its discovery by Tillet in 1930, CRP had been widely used in clinical settings as an indicator of infection response (Cha et al., 2022). Numerous studies had confirmed the clinical significance of CRP in the early diagnosis of sepsis. It was deemed a significant predictor and risk element for unfavorable consequences in individuals with sepsis (Stocker et al., 2021; Wang et al., 2013). In sepsis, anti-inflammatory cytokines were also discharged into the bloodstream, inducing immunosuppression and resulting in lymphocyte apoptosis (Heffernan et al., 2012; Menges et al., 1999; Hotchkiss et al., 2005). Lymphocytes, the primary white blood cells responsible for fighting infection and disease, played a crucial role (Grossman and Paul, 2015; Mastrogiovanni et al., 2022). Several studies had reported that lymphocytopenia, a reduced lymphocyte count was frequently observed in sepsis patients and is associated with unfavorable outcomes (Boomer et al., 2012; Drewry et al., 2014; Chung et al., 2015). At present, evaluating the prognosis of sepsis by combining multiple biochemical markers was an important focus in sepsis research (Huang et al., 2020; Can et al., 2018; Zhang et al., 2022). Studies had demonstrated that the lymphocyte-to-C-reactive protein ratio (LCR), which integrated lymphocyte and C-reactive protein levels, not only reflected the inflammatory status but also indicated immune function (Yildirim and Koca, 2021; Aoyama et al., 2022; Utsumi et al., 2022). LCR showed improved consistency compared to using lymphocyte and C-reactive protein individually, thereby enhancing the sensitivity in assessing the inflammatory status. Therefore, LCR had emerged as a valuable biomarker for the early detection and prediction of severe conditions such as coronavirus disease 2019 (COVID-19) and sepsis (Ullah et al., 2020; Li et al., 2023; Liu and Mu, 2023). However, the precise role of LCR in forecasting adverse outcomes in sepsis patients had yet to be fully understood. Therefore, the aim of this study was to examine whether LCR could serve as a novel indicator of inflammation, linked to both 30-day mortality and the incidence of acute kidney injury (AKI), among sepsis patients in the intensive care unit (ICU).

## Methods

### Study population

**FIGURE 1:**
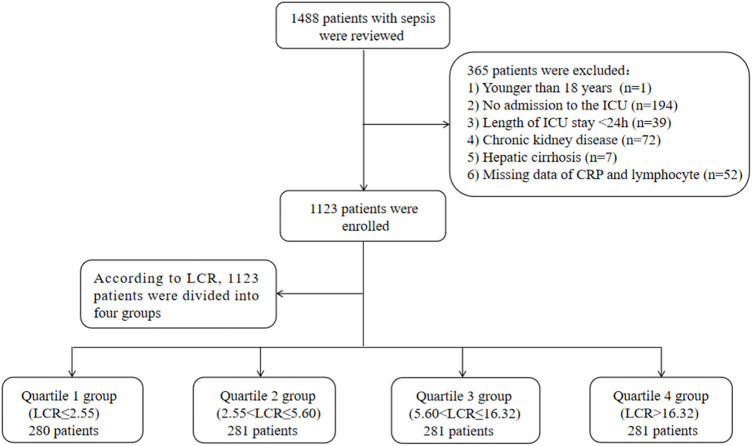
Flow of included patients through the trial. Abbreviations: LCR, Lymphocyte-to-C-Reactive Protein ratio; ICU, Intensive Care Unit.

This retrospective study was carried out at the Affiliated Hospital of Jiangsu University in Zhenjiang, China, spanning from January 2015 to November 2023. Data from 1,488 patients were extracted from the electronic medical record system. Sepsis was defined according to the Third International Consensus Definitions for Sepsis (Sepsis-3) (Singer et al., 2016), which specified that it was characterized by a sequential organ failure assessment (SOFA) score of ≥2 within 24 h of admission, along with at least one site of infection. Information on initial admissions for patients aged 18 years or older was gathered for this research. Additionally, the study excluded individuals who were not admitted to the ICU, those with an ICU stay of less than 24 h, individuals with a pre-existing chronic kidney disease, and those diagnosed with hepatic cirrhosis. Patients with missing data on CRP and lymphocyte on the first day of ICU admission were also excluded. Following a thorough evaluation, a total of 1,123 patients were included in the research and divided into four groups according to LCR quartiles (Figure 1). The study was designed in accordance with the Helsinki Declaration for studies involving humans. Approval for the study protocol was granted by the Affiliated Hospital of Jiangsu University (No. KY 2023K1007). Written informed consent was obtained from all participants involved.

### Variable extraction

The potential variables for this study were extracted from the electronic medical record system and can be categorized into six main groups: demographic information, comorbidities, infection pathogens, laboratory indicators, severity of illness scores, and treatments. A comprehensive list of the extracted variables was included in Table 1. Patient follow-up began upon admission and ended upon death or discharge. All laboratory indicators and illness severity scores were documented on the first day in the ICU. To avoid possible bias, variables were excluded if they had more than 20% missing values. Variables with missing data less than 20% were processed by multiple imputation using a random forest algorithm (Blazek et al., 2021; Austin et al., 2021). The value of LCR was calculated as [1,000*lymphocyte count (×10^9^ cells/L)]/[CRP (mg/L)]. Subsequently, patients were divided into four groups based on their LCR quartile ranges: Q1 (LCR ≤ 2.55, ≤ 25th percentile), Q2 (2.55 < LCR ≤ 5.60, 25th–50th percentile), Q3 (5.60 < LCR ≤ 16.32, 50th–75th percentile), and Q4 (LCR > 16.32, > 75th percentile).

**TABLE 1:** Covariates extracted in detail from the electronic medical record system.

Items	Composition
Demographic information	Age, gender, BMI, and smoking
Comorbidities	Hypertension, diabetes, coronary artery disease, COPD, and cerebral infarction
Infection pathogens	Gram-positive bacterial infection, Gram-negative bacterial infection, fungal infection, and viral infection
Laboratory indicators	WBC, Neu, Lym, Mon, Hb, PLT, CRP, Tbil, ALT, AST, albumin, glucose, creatinine, BUN, uric acid, D-dimer, potassium, and lactate
Severity of illness scores	APACHE II score and SOFA score
Treatments	CRRT, vasoactive drugs, and invasive ventilation

Abbreviations: BMI, body mass index; COPD, chronic obstructive pulmonary disease; WBC, white blood cell; Neu, neutrophil; Lym, lymphocyte; Mon, monocyte; Hb, hemoglobin; PLT, platelet; CRP, C-reactive protein; Tbil, total bilirubin; ALT, alanine transaminase; AST, aspartate aminotransferase; BUN, blood urea nitroge; APACHE II, Acute Physiology and Chronic Health Evaluation II; SOFA, sequential organ failure assessment; CRRT, continuous renal replacement therapy.

### Primary endpoint and secondary endpoint

The primary endpoint of the current study was 30-day mortality, while the secondary endpoint was the occurrence of AKI in the ICU. AKI was defined according to the 2012 Kidney Disease (Kellum et al., 2013): Improving Global Outcomes Clinical Practice Guidelines (KDIGO) as an increase in serum creatinine of 0.3 mg/dL within 48 h, a rise of at least 1.5 times the baseline level within the preceding 7 days, or a decrease in urine output to less than 0.5 mL/kg/h for more than 6 h. A diagnosis of AKI can be made if one or more of these criteria were met.

### Statistical analysis

The PASS software was used to calculate the test’s effectiveness. A significance level (α) of 0.05 was set, with a total sample size of 1,123, resulting in a power of 92.6% to analyze the relationship between LCR and patient outcomes. The statistical analyses were performed with SPSS software version 26.0, and the figures were created using GraphPad Prism 10.0. Data that had a normal distribution were expressed as means ± standard deviations and analyzed using independent t-tests or one-way analysis of variance. Non-normally distributed data were presented as medians and interquartile ranges and were analyzed using the Mann-Whitney U-test. Categorical variables were represented as percentages and were assessed using chi-square tests. Kaplan-Meier survival analysis was utilized to examine the connection between the clinical outcomes and LCR, both as a continuous variable and in quartiles, to determine the incidence rate of clinical outcome events among groups with varying LCR levels. Differences among groups were evaluated using the log-rank test. In addition, we employed receiver operating characteristic (ROC) analysis to estimate the predictive value of the LCR for 30-day mortality and the incidence of AKI. To estimate the hazard ratio (HR) and 95% confidence interval (CI) for the risk of LCR on outcomes, Cox proportional hazards and logistic regression models were employed. The LCR was incorporated into the models as either a continuous variable or a categorical variable. Further stratified analyses were performed according to age, gender, hypertension, diabetes, smoking, and lactate level to assess the consistency of the prognostic impact of LCR on outcomes. The interaction between LCR and the stratified variables was also tested. A significance level of *P* < 0.05 (two-sided) was deemed statistically significant for all analyses.

## Results

### Study population

The study included 1,123 patients diagnosed with sepsis. Table 2 provided an overview of the initial characteristics of these patients. The median age of the enrolled patients was 75 (IQR: 65–84) years. Among the patients, 707 (63.0%) were male, and the median BMI was 22.49 (IQR: 20.08–25.21) kg/m^2^. In terms of comorbidities, 579 (51.3%) patients had hypertension, 309 (27.5%) had diabetes, 161 (14.3%) had cerebral infarction, 116 (10.3%) had coronary artery disease, and 87 (7.7%) had COPD. Upon admission to the ICU, the median levels of Lym and CRP were 0.6 (IQR: 0.3–0.9) * 10^9^/L and 104.2 (IQR: 42.0–163.2) mg/L, respectively. The median LCR was 5.62 (IQR: 2.56–16.35). The mortality rate within 30 days was 28.1%, while the incidence of AKI was 45.6%. Please refer to Table 2 for additional variables and details.

**TABLE 2:** Characteristics and outcomes of participants categorized by LCR.

Variables	Overall	Q1 group (LCR ≤ 2.55)	Q2 group (2.55 < LCR ≤ 5.60)	Q3 group (5.60 < LCR ≤ 16.32)	Q4 group (LCR > 16.32)	*P*-value
N	1,123	280	281	281	281	-
Age, years	75 (65–84)	76 (65–84)	75 (65–83)	77 (67–85)	74 (64–85)	0.230
Male, n (%)	707 (63.0)	169 (60.4)	189 (67.3)	183 (65.1)	166 (59.1)	0.143
BMI, kg/m^2^	22.49 (20.08–25.21)	22.33 (20.08–24.65)	22.72 (20.41–25.37)	22.49 (19.95–25.71)	22.49 (19.99–25.26)	0.575
Smoking, n (%)	229 (20.4)	62 (22.1)	72 (25.7)	47 (16.7)	48 (17.1)	0.022
Comorbidities, n (%)						
Hypertension	579 (51.3)	135 (48.2)	145 (51.6)	162 (57.7)	137 (48.8)	0.097
Diabetes	309 (27.5)	73 (26.1)	86 (30.6)	75 (26.7)	75 (26.7)	0.609
Coronary artery disease	116 (10.3)	26 (9.3)	40 (14.2)	28 (10.0)	22 (7.9)	0.077
COPD	87 (7.7)	14 (5.0)	17 (6.0)	27 (9.6)	29 (10.3)	0.045
Cerebral infarction	161 (14.3)	33 (11.8)	41 (14.6)	47 (16.7)	40 (14.2)	0.422
Infection pathogens, n (%)						
Gram-positive bacteria	136 (12.1)	36 (12.9)	34 (12.1)	33 (11.7)	33 (11.7)	<0.001
Gram-negative bacteria	335 (29.8)	90 (32.1)	99 (35.2)	82 (29.2)	64 (22.8)	0.975
Fungus	77 (6.9)	21 (7.5)	25 (8.9)	23 (8.2)	8 (2.8)	0.020
Virus	60 (5.3)	60 (5.3)	18 (6.4)	15 (5.3)	11 (3.9)	0.607
Laboratory tests						
WBC *10^9^/L	11.4 (7.4–17.1)	10.4 (6.6–15.8)	12.7 (8.0–18.3)	11.4 (8.0–17.4)	11.0 (7.3–16.2)	0.003
Neu *10^9^/L	10.1 (6.3–15.5)	9.6 (6.1–15.1)	11.9 (6.9–16.9)	9.8 (6.8–15.4)	9.3 (6.0–14.4)	0.002
Lym *10^9^/L	0.6 (0.3–0.9)	0.3 (0.2–0.4)	0.5 (0.4–0.7)	0.8 (0.5–1.1)	0.9 (0.6–1.4)	<0.001
Mon *10^9^/L	0.4 (0.2–0.7)	0.3 (0.1–0.4)	0.4 (0.2–0.7)	0.5 (0.3–0.7)	0.5 (0.2–0.8)	<0.001
Hb, g/dL	115 (97–130)	111 (93–125)	112 (97–130)	115 (97–129)	120 (105–138)	<0.001
PLT *10^9^/L	149 (95–214)	117 (77–171)	136 (86–205)	154 (103–227)	189 (141–254)	<0.001
CRP, mg/L	104.2 (42.0–163.2)	182.8 (136.4–240.0)	139.6 (97.0–182.1)	86.7 (54.7–117.6)	12.3 (4.1–31.3)	<0.001
LCR	5.62 (2.56–16.35)	1.67 (1.14–2.08)	3.91 (3.09–4.65)	9.16 (7.21–11.51)	64.71 (29.01–197.56)	<0.001
Tbil, μmol/L	17.4 (10.9–28.2)	19.0 (12.1–32.2)	19.2 (12.4–32.7)	16.8 (10.7–26.3)	14.0 (8.4–23.2)	<0.001
ALT, U/L	32.0 (21.0–56.0)	33.0 (22.0–60.8)	36.0 (21.2–62.5)	30.6 (21.0–51.5)	31.0 (20.1–50.0)	0.211
AST, U/L	38.1 (23.9–73.0)	47.5 (26.0–85.5)	41.0 (25.0–90.2)	35.0 (23.0–65.0)	32.0 (21.0–62.0)	<0.001
Albumin, g/L	28.2 (24.2–33.2)	27.0 (22.7–31.0)	27.4 (23.9–32.7)	27.6 (24.3–32.5)	31.6 (27.3–36.4)	<0.001
Glucose, mmol/L	8.2 (6.6–11.8)	8.6 (6.6–12.4)	8.8 (6.7–12.7)	7.9 (6.4–11.4)	7.9 (6.5–10.1)	0.015
Creatinine, μmol/L	92.6 (63.7–153.1)	119.6 (73.6–200.0)	110.4 (67.5–165.3)	86.7 (61.1–134.7)	76.1 (56.5–122.1)	<0.001
BUN, mmol/L	8.89 (6.04–13.95)	11.33 (7.00–18.46)	10.18 (6.72–14.90)	8.04 (5.67–11.66)	7.11 (5.18–10.28)	<0.001
Uric acid, μmol/L	286.9 (192.3–411.7)	305.4 (205.3–471.0)	286.5 (193.3–414.6)	286.0 (187.3–395.4)	274.4 (189.2–384.7)	0.026
D-dimer, mg/L	4.2 (2.1–8.4)	5.1 (2.7–9.6)	5.1 (2.5–8.9)	3.8 (2.0–7.5)	3.4 (1.6–7.5)	<0.001
Potassium, mmol/L	3.7 (3.3–4.2)	3.7 (3.3–4.2)	3.7 (3.3–4.2)	3.6 (3.2–4.0)	3.8 (3.3–4.2)	0.041
Lactate, mmol/L	2.1 (1.4–3.6)	2.2 (1.6–3.8)	2.1 (1.4–3.5)	2.1 (1.3–3.9)	2.0 (1.3–3.4)	0.016
Severity scoring						
APACHE II score	25 (19–30)	26 (20–32)	25 (20–30)	25 (19–30)	25 (18–30)	0.121
SOFA score	12 (10–14)	12 (9–14)	12 (9–14)	12 (10–14)	13 (10–15)	<0.001
Treatments						
CRRT, n (%)	78 (6.9)	28 (10.0)	21 (7.5)	17 (6.0)	12 (4.3)	0.054
Vasoactive drug, n (%)	748 (66.6)	224 (80.0)	197 (70.1)	178 (63.3)	149 (53.0)	<0.001
Invasive ventilation, n (%)	752 (67.0)	188 (67.1)	179 (63.7)	186 (66.2)	199 (70.8)	0.345
Endpoints						
30-day mortality, n (%)	316 (28.1)	103 (36.8)	93 (33.1)	74 (26.3)	46 (16.4)	<0.001
AKI, n (%)	512 (45.6)	165 (58.9)	141 (50.2)	117 (41.6)	89 (31.7)	<0.001
Length of ICU stay, days	6 (3–12)	5 (2–11)	6 (3–11)	7 (4–13)	6 (3–12)	<0.001
Length of hospital stay, days	16 (11–25)	17 (11–25)	16 (10–24)	17 (11–27)	17 (9–27)	0.240
60-day mortality, n (%)	375 (33.4)	118 (42.1)	108 (38.4)	91 (32.4)	58 (20.6)	<0.001
ICU mortality, n (%)	358 (31.9)	114 (40.7)	103 (36.7)	86 (30.6)	55 (19.6)	<0.001
Hospital mortality, n (%)	379 (33.7)	120 (42.9)	109 (38.8)	91 (32.4)	59 (21.0)	<0.001

Abbreviations: LCR, Lymphocyte-to-C-Reactive Protein ratio; BMI, body mass index; COPD, chronic obstructive pulmonary disease; WBC, white blood cell; Neu, neutrophil; Lym, lymphocyte; Mon, monocyte; Hb, hemoglobin; PLT, platelet; CRP, C-reactive protein; Tbil, total bilirubin; ALT, alanine transaminase; AST, aspartate aminotransferase; BUN, blood urea nitroge; APACHE II, Acute Physiology and Chronic Health Evaluation II; SOFA, sequential organ failure assessment; CRRT, continuous renal replacement therapy; AKI, acute kidney injury; ICU, intensive care unit.

### Baseline characteristics

In Table 2, the baseline characteristics were divided into quartiles according to the LCR. The median values of LCR for each quartile were as follows: 1.67 (IQR: 1.14–2.08), 3.91 (IQR: 3.09–4.65), 9.16 (IQR: 7.21–11.51), and 64.71 (IQR: 29.01–197.56). Patients with higher LCR demonstrated a lower prevalence of smoking, Gram-positive bacterial and fungal infections, lower levels of WBC, Neu, CRP, Tbil, AST, glucose, creatinine, BUN, uric acid, and lactate. On the other hand, they exhibited higher levels of Lym, Mon, Hb, PLT, and albumin, as well as a higher severity of SOFA score. Additionally, patients in the higher LCR group had a lower proportion of vasoactive drug usage compared to the lower LCR group. With an increase in LCR, there was a gradual decrease in the following: 30-day mortality rate (36.8% vs. 33.1% vs. 26.3% vs. 16.4%, *P* < 0.001), occurrence of AKI (58.9% vs. 50.2% vs. 41.6% vs. 31.7%, *P* < 0.001), length of stay in the ICU (5 days vs. 6 days vs. 7 days vs. 6 days, *P* < 0.001), 60-day mortality rate (42.1% vs. 38.4% vs. 32.4% vs. 20.6%, *P* < 0.001), ICU mortality rate (40.7% vs. 36.7% vs. 30.6% vs. 19.6%, *P* < 0.001), and hospital mortality rate (42.9% vs. 38.8% vs. 32.4% vs. 21.0%, *P* < 0.001). Due to the link between the Q4 group and low 30-day mortality, a further comparison was conducted between Q4 and the combined first to third quartiles (Q1-3). This analysis revealed that different grouping approaches yielded similar results (Supplementary Table S1).

**FIGURE 2:**
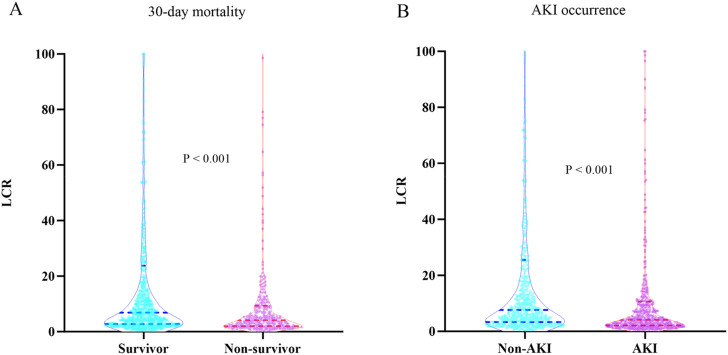
**(A)** Boxplots of the LCR showing the distribution in the Survivor group and Non-survivor group at 30 days. **(B)** Boxplots of the LCR showing the distribution in the Non-AKI group and AKI group. Abbreviations: LCR, Lymphocyte-to-C-Reactive Protein ratio; AKI, Acute kidney injury.

Table 3 presented the variances in baseline characteristics between survivors and non-survivors over their 30-day hospital stay. Non-survivors tended to be older and had a lower BMI. They also had a higher prevalence of coronary artery disease, COPD, cerebral infarction, fungal and viral infections. In terms of laboratory values, Non-survivors had higher counts of WBC, Neu, CRP, Tbil, ALT, AST, creatinine, BUN, uric acid, D-dimer, and potassium. Additionally, they had elevated levels of lactate and lower levels of Lym, Hb, PLT, and albumin. Non-survivors also had higher APACHE II scores and a greater likelihood of receiving CRRT, vasoactive drugs, and invasive ventilation. Significantly, the levels of LCR were higher in the Non-survivor group compared to the Survivor group (6.84 vs. 4.09, *P* < 0.001). Figure 2A depicted the distribution of LCR categorized by the 30-day in-hospital mortality status.

**TABLE 3:** Characteristics and outcomes of participants categorized by LCR.

Variables	Survivor	Non-survivor	*P*-value	Non-AKI	AKI	*P*-value
N	807	316	-	611	512	-
Age, years	74 (62–83)	79 (72–86)	<0.001	75 (63–85)	76 (66–84)	0.336
Male, n (%)	505 (62.6)	202 (63.9)	0.674	390 (63.8)	317 (61.9)	0.508
BMI, kg/m^2^	22.76 (20.31–25.62)	21.87 (19.56–24.22)	<0.001	22.49 (20.07–24.97)	22.49 (20.20–25.40)	0.639
Smoking, n (%)	152 (18.8)	77 (24.4)	0.036	134 (21.9)	95 (18.6)	0.167
Comorbidities, n (%)						
Hypertension	406 (50.3)	173 (54.7)	0.181	301 (49.3)	278 (54.3)	0.093
Diabetes	219 (27.1)	90 (28.5)	0.650	146 (23.9)	163 (31.8)	0.003
Coronary artery disease	68 (8.4)	48 (15.2)	0.001	46 (7.5)	70 (13.7)	0.001
COPD	49 (6.1)	38 (12.0)	0.001	51 (8.3)	36 (7.0)	0.411
Cerebral infarction	104 (12.9)	57 (18.0)	0.027	86 (14.1)	75 (14.6)	0.785
Infection pathogens, n (%)						
Gram-positive bacteria	93 (11.5)	43 (13.6)	0.336	73 (11.9)	63 (12.3)	0.855
Gram-negative bacteria	237 (29.4)	98 (31.0)	0.588	153 (25.0)	182 (35.5)	<0.001
Fungus	41 (5.1)	36 (11.4)	<0.001	27 (4.4)	50 (9.8)	<0.001
Virus	27 (3.3)	33 (10.4)	<0.001	42 (6.9)	18 (3.5)	0.013
Laboratory tests						
WBC *10^9^/L	11.1 (7.3–16.8)	12.6 (7.9–17.9)	0.024	10.5 (7.0–15.4)	12.9 (8.0–18.9)	<0.001
Neu *10^9^/L	9.7 (6.2–15.2)	11.5 (7.0–16.6)	0.005	9.2 (6.1–14.0)	11.8 (6.8–17.4)	<0.001
Lym *10^9^/L	0.6 (0.4–1.0)	0.4 (0.3–0.7)	<0.001	0.6 (0.4–0.9)	0.5 (0.3–0.8)	0.001
Mon *10^9^/L	0.4 (0.2–0.7)	0.4 (0.2–0.7)	0.329	0.4 (0.2–0.6)	0.4 (0.2–0.7)	0.965
Hb, g/dL	117 (100–132)	107 (88–126)	<0.001	117 (100–131)	112 (93–129)	0.002
PLT *10^9^/L	161 (105–228)	122 (74–190)	<0.001	167 (115–233)	128 (78–192)	<0.001
CRP, mg/L	102.5 (33.8–160.9)	111 (58–172)	0.006	84.3 (31.5–149.8)	129.3 (61.1–186.7)	<0.001
LCR	6.84 (2.70–23.74)	4.09 (1.99–9.37)	<0.001	7.65 (3.30–25.48)	4.10 (2.07–10.59)	<0.001
Tbil, μmol/L	16.9 (10.8–27.4)	18.4 (11.2–30.1)	0.096	15.8 (10.0–23.3)	20.9 (12.2–34.4)	<0.001
ALT, U/L	31.0 (21.0–50.0)	36.0 (22.0–74.0)	0.003	29.0 (20.0–49.0)	36.1 (23.0–76.1)	<0.001
AST, U/L	35.0 (22.0–64.0)	52.5 (28.3–124.0)	<0.001	33.0 (22.0–57.3)	52.0 (27.0–125.8)	<0.001
Albumin, g/L	28.9 (24.5–33.6)	27.0 (23.4–32.2)	0.002	29.5 (24.9–33.5)	27.2 (23.3–32.7)	<0.001
Glucose, mmol/L	8.1 (6.6–11.4)	8.6 (6.6–12.2)	0.093	7.9 (6.4–10.7)	8.8 (6.7–13.1)	<0.001
Creatinine, μmol/L	85.0 (59.7–133.4)	126.2 (72.9–191.8)	<0.001	66.0 (52.2–81.1)	160.0 (126.4–239.0)	<0.001
BUN, mmol/L	8.20 (5.55–11.69)	11.90 (7.65–19.80)	<0.001	6.53 (4.87–8.89)	13.75 (9.70–20.44)	<0.001
Uric acid, μmol/L	275.1 (185.1–377.7)	341.7 (214.2–509.2)	<0.001	210.3 (150.0–291.5)	401.7 (299.6–524.7)	<0.001
D-dimer, mg/L	3.8 (2.0–7.2)	6.5 (2.9–11.7)	<0.001	3.4 (1.8–6.4)	6.1 (2.9–10.8)	<0.001
Potassium, mmol/L	3.7 (3.3–4.1)	3.8 (3.4–4.4)	0.003	3.6 (3.3–4.0)	3.8 (3.3–4.5)	<0.001
Lactate, mmol/L	2.0 (1.3–3.0)	2.9 (2.0–5.3)	<0.001	1.8 (1.3–2.7)	2.6 (1.8–5.0)	<0.001
Severity scoring						
APACHE II score	24 (18–29)	28 (23–34)	<0.001	24 (18–29)	27 (21–33)	<0.001
SOFA score	12 (9–14)	13 (11–15)	0.108	11 (9–14)	13 (11–15)	<0.001
Treatments						
CRRT, n (%)	30 (3.7)	48 (15.2)	<0.001	3 (0.5)	75 (14.6)	<0.001
Vasoactive drug, n (%)	466 (57.7)	282 (89.2)	<0.001	342 (56.0)	406 (79.3)	<0.001
Invasive ventilation, n (%)	472 (58.5)	280 (88.6)	<0.001	413 (67.6)	339 (66.2)	<0.001
Endpoints						
30-day mortality, n (%)	-	-	-	121 (19.8)	195 (38.1)	<0.001
AKI, n (%)	317 (39.3)	195 (61.7)	<0.001	-	-	-
Length of ICU stay, days	5 (3–10)	8 (4–14)	0.572	5 (3–11)	7 (4–12)	<0.001
Length of hospital stay, days	19 (12–29)	12 (6–19)	0.886	17 (11–26)	16 (9–25)	0.014
60-day mortality, n (%)	-	-	-	152 (24.9)	223 (43.6)	<0.001
ICU mortality, n (%)	-	-	-	146 (23.9)	212 (41.4)	<0.001
Hospital mortality, n (%)	-	-	-	156 (25.5)	223 (43.6)	<0.001

Abbreviations: LCR, Lymphocyte-to-C-Reactive Protein ratio; BMI, body mass index; COPD, chronic obstructive pulmonary disease; WBC, white blood cell count; Neu, neutrophil; Lym, lymphocyte; Mon, monocyte; Hb, hemoglobin; PLT, platelet; CRP, C-reactive protein; Tbil, total bilirubin; ALT, alanine transaminase; AST, aspartate aminotransferase; BUN, blood urea nitroge; APACHE II, Acute Physiology and Chronic Health Evaluation II; SOFA, sequential organ failure assessment; CRRT, continuous renal replacement therapy; AKI, acute kidney injury; ICU, intensive care unit.

### Association between the 30-day mortality and LCR

**FIGURE 3:**
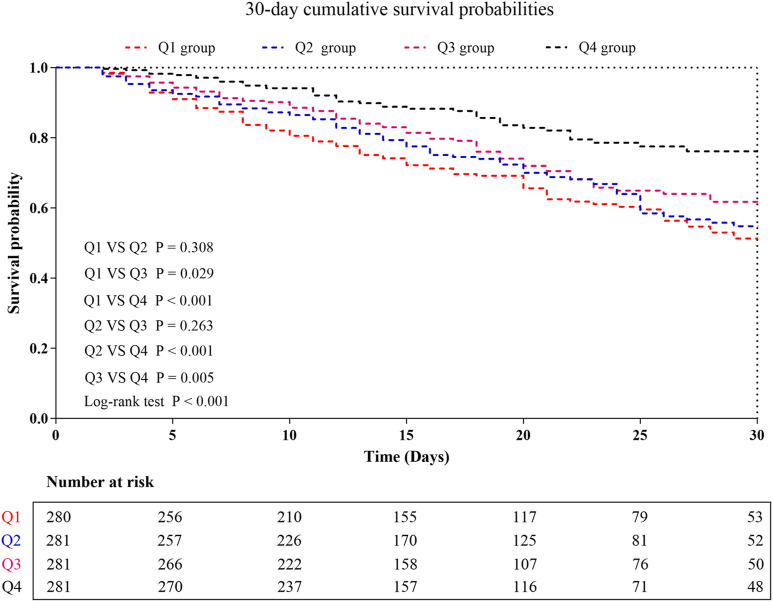
Kaplan–Meier curves showing cumulative probability of 30-day mortality. LCR quartiles: Q1 group (LCR ≤ 2.55); Q2 group (2.55 < LCR ≤ 5.60); Q3 group (5.60 < LCR ≤ 16.32); Q4 group (LCR > 16.32).

**FIGURE 4:**
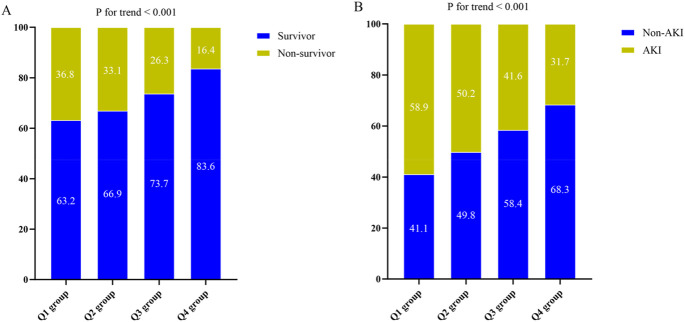
**(A)** The prevalence of 30-day mortality ratio among different quartiles of LCR. **(B)** The prevalence of AKI occurrence ratio among different quartiles of LCR. LCR quartiles: Q1 group (LCR ≤ 2.55); Q2 group (2.55 < LCR ≤ 5.60); Q3 group (5.60 < LCR ≤ 16.32); Q4 group (LCR > 16.32). Abbreviations: LCR, Lymphocyte-to-C-Reactive Protein ratio; AKI, Acute kidney injury.

**FIGURE 5:**
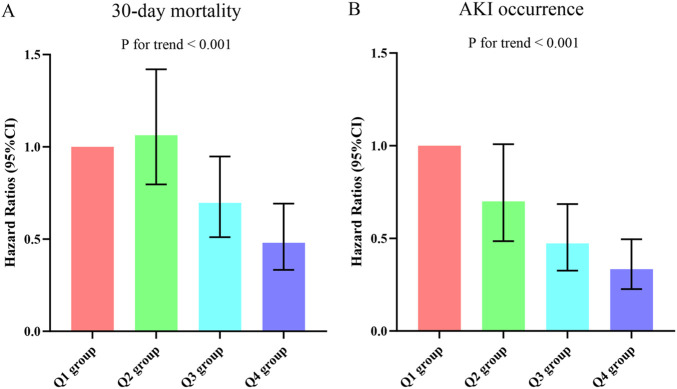
Hazard ratios (95% CIs) for 30-day mortality **(A)** and AKI occurrence **(B)** according to LCR quartiles after adjusting for age, gender, BMI, hypertension, diabetes, WBC, Neu, Hb, lactate, APACHE II score, SOFA score, and invasive ventilation. Error bars indicate 95% CIs. The first quartile is the reference. LCR quartiles: Q1 group (LCR ≤ 2.55); Q2 group (2.55 < LCR ≤ 5.60); Q3 group (5.60 < LCR ≤ 16.32); Q4 group (LCR > 16.32). LCR, Lymphocyte-to-C-Reactive Protein ratio; BMI, body mass index; WBC, white blood cell; Neu, neutrophil; Hb, hemoglobin; APACHE II, Acute Physiology and Chronic Health Evaluation II; SOFA, Sequential Organ Failure Assessment; AKI, Acute kidney injury.

Based on the Kaplan-Meier survival analysis curves, it was observed that patients with higher LCR levels had a reduced risk of 30-day mortality (Figure 3). Additionally, Figure 4A indicated a decreasing trend in mortality rates with higher LCR. Furthermore, our analysis demonstrated that the LCR had a higher predictive power for 30-day mortality (AUC: 0.620: 95% CI: 0.585–0.656; *P* < 0.001) compared to the other indicators, such as WBC, Neu, lymphocyte, CRP, albumin, APACHE II score, and SOFA score (Supplementary Table S2). Supplementary Table S3 displayed the outcomes of the univariate COX regression analysis for the risk of 30-day mortality in sepsis patients. Significant variables from the univariate analysis (*P* < 0.05) were included, along with factors recommended by clinicians based on their expertise, as independent variables for the COX regression analysis. The influential factors that were identified included LCR, age, BMI, Neu, Hb, lactate, APACHE II score, and invasive ventilation. To investigate the association between LCR and 30-day mortality, multivariate Cox proportional risk analysis was conducted. The results indicated that LCR was a notable risk factor in the unadjusted model [HR (95%CI): 0.351 (0.131–0.938); *P* = 0.037], partly adjusted model [HR (95%CI): 0.354 (0.130–0.969); *P* = 0.043], and fully adjusted model [HR (95%CI): 0.370 (0.142–0.963); *P* = 0.042] when LCR was treated as a continuous variable. When LCR was considered as a categorical variable, patients in the higher quartile of LCR had a substantially lower risk of 30-day mortality compared to those in the lowest quartile, as observed in the three established Cox proportional hazards models: unadjusted model [HR (95%CI): 0.446 (0.315–0.631); *P* < 0.001], partly adjusted model [HR (95%CI): 0.443 (0.311–0.631); *P* < 0.001], and fully adjusted model [HR (95%CI): 0.480 (0.333–0.692); *P* < 0.001] (Table 4). Additionally, there was a downward tendency for the risk of 30-day mortality to increase with the LCR, as shown in Figure 5A.

**TABLE 4:** Cox proportional hazards and logistic regression models for 30-day mortality and AKI occurrence.

Variables	Model 1			Model 2			Model 3		
	HR (95% CI)	*P*-value	P for trend	HR (95% CI)	*P*-value	P for trend	HR (95% CI)	*P*-value	P for trend
30-day mortality									
Continuous variable per unit	0.351 (0.131–0.938)	0.037		0.354 (0.130–0.969)	0.043		0.370 (0.142–0.963)	0.042	
Quartile^a^			<0.001			<0.001			<0.001
Q1 group	Ref			Ref			Ref		
Q2 group	0.986 (0.745–1.305)	0.921		1.041 (0.783–1.383)	0.784		1.063 (0.796–1.420)	0.678	
Q3 group	0.678 (0.502–0.915)	0.011		0.719 (0.531–0.974)	0.033		0.696 (0.510–0.948)	0.022	
Q4 group	0.446 (0.315–0.631)	<0.001		0.443 (0.311–0.631)	<0.001		0.480 (0.333–0.692)	<0.001	
AKI occurrence									
Continuous variable per unit	0.484 (0.274–0.856)	0.013		0.524 (0.299–0.918)	0.024		0.541 (0.307–0.953)	0.034	
Quartile^a^			<0.001			<0.001			<0.001
Q1 group	Ref			Ref			Ref		
Q2 group	0.702 (0.503–0.980)	0.038		0.623 (0.441–0.880)	0.007		0.699 (0.485–1.008)	0.055	
Q3 group	0.497 (0.355–0.696)	<0.001		0.456 (0.322–0.646)	<0.001		0.473 (0.326–0.685)	<0.001	
Q4 group	0.323 (0.229–0.457)	<0.001		0.320 (0.223–0.458)	<0.001		0.334 (0.226–0.495)	<0.001	

Model 1: unadjusted.

Model 2: adjusted for age, gender, BMI, hypertension, diabetes, WBC, neu, and invasive ventilation.

Model 3: adjusted for age, gender, BMI, hypertension, diabetes, WBC, neu, Hb, lactate, APACHE II, score, SOFA, score, and invasive ventilation.

^a^ LCR: Q1 group (LCR ≤ 2.55); Q2 group (2.55 < LCR ≤ 5.60); Q3 group (5.60 < LCR ≤ 16.32); Q4 group (LCR > 16.32).

Abbreviations: LCR, Lymphocyte-to-C-Reactive Protein ratio; BMI, body mass index; WBC, white blood cell; Neu, neutrophil; Hb, hemoglobin; APACHE II, Acute Physiology and Chronic Health Evaluation II; SOFA, sequential organ failure assessment; AKI, acute kidney injury.

**FIGURE 6:**
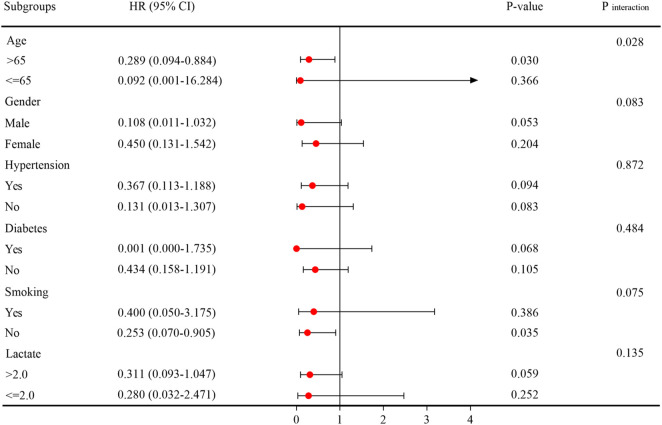
Subgroup analysis regarding the influence of different LCR in the 30-day mortality. Abbreviations: LCR, Lymphocyte-to-C-Reactive Protein ratio.

Additionally, we conducted a further analysis of the risk stratification value of LCR for 30-day mortality in various subgroups of the enrolled patients, including age, gender, hypertension, diabetes, smoking, and lactate level. Among sepsis patients, the LCR was found to be significantly associated with a higher risk of 30-day mortality in two subgroups: those aged over 65 years [HR (95%CI): 0.289 (0.094–0.884); *P* = 0.030] and non-smokers [HR (95%CI): 0.253 (0.070–0.905); *P* = 0.035]. Interestingly, it was observed that the predictive value of LCR appeared to be more pronounced in patients aged > 65 years compared to those aged ≤ 65 years [HR (95%CI) aged > 65 years 0.289 (0.094–0.884) vs. Aged ≤ 65 years 0.092 (0.001–16.284); *P* for interaction = 0.028] (Figure 6).

### Relationship between LCR and AKI occurrence

The incidence of AKI varied significantly across the LCR groups (Q1: 58.9% vs. Q2: 50.2% vs. Q3: 41.6% vs. Q4: 31.7%, *P* < 0.001) (Table 2), suggesting a decreasing trend in AKI occurrence with higher LCR levels (Figure 4B). Additionally, the AUC for the LCR in discriminating AKI occurrence was 0.625 (95%CI: 0.592–0.657, *P* < 0.001). The cut-off value for the LCR to predict AKI occurrence was determined to be 4.49 (Supplementary Table S2). Table 3 displayed the differences in baseline characteristics between patients without AKI and those who developed AKI during their ICU stay. The AKI group had a higher prevalence of diabetes, coronary artery disease, Gram-negative bacteria, fungus, and virus infections. Furthermore, they exhibited elevated levels of WBC, Neu, CRP, LCR, Tbil, ALT, AST, glucose, creatinine, uric acid, D-dimer, and potassium, as well as lower levels of Lym, Hb, PLT, and albumin. Additionally, the AKI group had higher severity scores such as APACHE II score and SOFA score and a higher proportion of patients receiving CRRT, vasoactive drugs, and invasive ventilation. Compared to individuals without AKI, those with AKI had higher rates of 30-day mortality, 60-day mortality, ICU mortality, hospital mortality, longer ICU stays, and shorter hospital stays. Figure 2B illustrateed the distribution of the LCR according to AKI occurrence.

Supplementary Table S4 presented the findings of a binary logistic regression analysis that examined the association between the occurrence of AKI in sepsis patients and the LCR variable. In the analysis, which included variables from the univariate analysis, the results showed a significant association between LCR and AKI occurrence across all models. When LCR was considered as a continuous variable, the unadjusted model indicated a HR of 0.484 (95% CI: 0.274–0.856) with a *P*-value of 0.013. Similarly, the partially adjusted model yielded a HR of 0.524 (95% CI: 0.299–0.918) with a *p*-value of 0.024, and the fully adjusted model showed a HR of 0.541 (95% CI: 0.307–0.953) with a *p*-value of 0.034. When LCR was analyzed as a nominal variable, the risk of AKI occurrence displayed a decreasing trend with increasing LCR quartiles. Specifically, compared to the Q1 group, the risk of AKI occurrence was lower in the Q2 group, Q3 group, and Q4 group. The HR (95% CI) values for Q2 group, Q3 group, and Q4 group were 0.699 (0.485–1.008), 0.473 (0.326–0.685), and 0.334 (0.226–0.495) respectively (Table 4). This trend was found to be statistically significant, with a P-value for trend <0.001 (Figure 5B).

**FIGURE 7:**
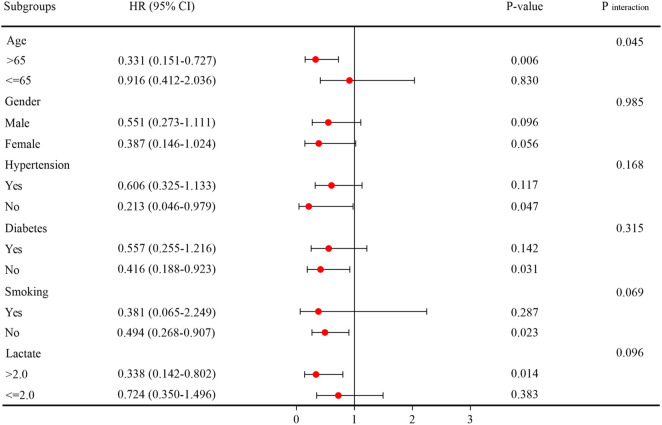
Subgroup analysis regarding the influence of different LCR in the AKI occurrence. Abbreviations: LCR, Lymphocyte-to-C-Reactive Protein ratio; AKI, Acute kidney injury.

Additionally, we conducted stratified analyses to explore the relationship between LCR and AKI occurrence based on potential modifiers, including age, gender, hypertension, diabetes, smoking, and lactate level. These findings were presented in Figure 7. We found a significant association between LCR and an increased risk of AKI in a subgroup of those aged > 65 years [HR (95%CI): 0.331 (0.151–0.727); *P* = 0.006], those without hypertension [HR (95%CI): 0.213 (0.046–0.979); *P* = 0.047], those without diabetes [HR (95%CI): 0.416 (0.188–0.923); *P* = 0.031], those without smoking [HR (95%CI): 0.494 (0.268–0.907); *P* = 0.023], and those with lactate level > 2.0 mmol/L [HR (95%CI): 0.338 (0.142–0.802); *P* = 0.014]. Interestingly, the impact of LCR on AKI risk appeared more significant in patients aged > 65 years compared to those aged ≤ 65 years, as indicated by the HR of 0.331 (95%CI: 0.151–0.727) in the former group and 0.916 (95%CI: 0.412–2.036) in the latter group. This interaction was statistically significant, with a *P*-value for interaction of 0.045.

## Discussion

In this study, we had identified a specific correlation between the LCR and adverse outcomes in ICU patients with sepsis. Our findings indicated that a decreased LCR served as a strong and independent predictor of both 30-day mortality and the occurrence of AKI in ICU patients with sepsis. Notably, this association remained significant even after considering a wide range of clinical and laboratory variables. The results indicated that a lower LCR can be considered a novel indicator of poorer prognosis. Consequently, the LCR showed potential as a valuable tool for clinicians in making decisions and may be an independent risk factor for ICU patients with sepsis.

Sepsis had always posed a significant challenge in the clinical treatment of patients with severe infections (Grande et al., 2019). Clinical studies had demonstrated that early detection of sepsis and prompt targeted treatment of prognostic risk factors can greatly reduce mortality rates (Rhodes et al., 2017; Yin et al., 2018). Previous research had focused on examining the predictive accuracy of multiple biomarkers in assessing the prognosis of sepsis patients. These biomarkers included the neutrophil-to-lymphocyte ratio (NLR), plasma 7-ketocholesterol (7-KC), C-reactive protein-to-albumin ratio (CAR), serum S100 calcium-binding protein B (S100B), milk fat globule epidermal growth factor 8 (MFG-E8), and red blood cell distribution width (Huang et al., 2020; Liu et al., 2023a; Hu Z. D. et al., 2020; Zhang et al., 2023; Hu et al., 2023; Wu et al., 2022). Notably, the LCR had recently emerged as a novel prognostic indicator for various cancers and the severity assessment of COVID-19 (Ullah et al., 2020; Iseda et al., 2021; Takeuchi et al., 2021; Okugawa et al., 2020; Angin et al., 2021). However, the potential prognostic value of LCR in sepsis patients remained unexplored in existing literature.

Sepsis was characterized by a systemic inflammatory response triggered by infection (Singer et al., 2016). In addition to the inflammatory responses, compensatory anti-inflammatory responses also occured, resulting in the suppression of the host’s immune function (de Pablo et al., 2014). One prominent aspect of immunosuppression observed in experimental sepsis was lymphocyte apoptosis. This process further contributed to immunosuppression, making the host more vulnerable to invading pathogens (Hotchkiss et al., 2005; Lang and Matute-Bello, 2009; Chu et al., 2021). Sepsis patients often experienced a significant decrease in lymphocyte count, which can be attributed to lymphocyte marginalization, increased apoptosis, and cell redistribution (Felmet et al., 2005). Lymphocytopenia, a common condition observed in hospitalized patients with sepsis, had been linked to elevated mortality rates (Venet et al., 2010; Adrie et al., 2017). A meta-analysis indicated that individuals experiencing lymphocytopenia faced a threefold higher risk of developing severe sepsis (Zhao et al., 2020). A retrospective study conducted in Spain revealed that sepsis patients with concurrent lymphocytopenia exhibited increased rates of ICU admission and higher mortality rates (Cilloniz et al., 2021). Additionally, a study by Sheikh Motahar Vahedi et al. (2019) concluded that lymphopenia independently predicted 28-day mortality in sepsis patients. Even sepsis individuals with low-normal lymphocyte counts were found to have higher short-term mortality rates compared to those with higher lymphocyte counts.

Currently, the prevailing understanding of sepsis pathophysiology was based on the balance theory of host response. According to this theory, sepsis occured when there was an imbalance in immune responses, leading to an exaggerated inflammatory reaction characterized by the release of inflammatory factors. This systemic inflammation can then trigger multiple organ failure. Consequently, inflammatory factors served as prominent markers throughout the progression of sepsis (Chousterman et al., 2017). CRP, an acute-phase protein synthesized by the liver, exhibited increased levels in response to inflammation or infection (Black et al., 2004). Higher levels of CRP were found to be indicative of a more severe disease status and a poorer prognosis in patients with sepsis (Stocker et al., 2021; Gülcher et al., 2016). A study by Cha et al. (2022) involving 2,291 elderly patients diagnosed with sepsis and admitted to the emergency department found that combining CRP levels with other inflammatory biomarkers could predict 28-day mortality. Koozi et al. (2020) discovered that an admission CRP level greater than 100 mg/L was associated with 30-day mortality in sepsis patients. Furthermore, a prospective study involving 349 patients identified CRP as an independent predictive factor for short-term mortality in sepsis (Huang et al., 2022). That being said, it was worth noting that some studies had compared CRP levels between ICU survivors and non-survivors with sepsis but did not find any significant differences (Silvestre et al., 2009; Pettilä et al., 2002; Ryoo et al., 2019). This suggested that CRP alone may not be the sole determinant of sepsis prognosis, and additional factors should be considered in predicting outcomes.

Recently, there had been a growing interest in combining lymphocyte count and CRP level to enhance their predictive value as individual markers. The LCR, an index obtained by dividing the lymphocyte count by the CRP level, had emerged as a promising marker for systemic inflammation and had garnered increased attention. In a retrospective cohort study focusing on incarcerated hernias, it was found that a low preoperative LCR level could potentially serve as a biomarker for estimating intestinal ischemia (Yildirim et al., 2021). Liu et al. (2023b) suggested that preoperative LCR served as an innovative and valuable prognostic indicator for predicting the incidence of major adverse cardiovascular events (MACEs) during hospitalization and in the long term following primary percutaneous coronary intervention in patients diagnosed with ST-segment elevation myocardial infarction (STEMI). Yildirim and Koca (2021) investigated LCR as a prognostic biomarker in colorectal cancer, and discovered that LCR outperformed other prognostic factors in predicting postoperative complications in patients undergoing colorectal cancer surgery on day 5. Hu H. et al. (2020) also demonstrated that preoperative LCR could be used as an effective and independent prognostic indicator for patients with osteosarcoma. Moreover, elevated LCR had shown prognostic value in patients with esophageal cancer, gastric cancer, and hepatocellular carcinoma (Iseda et al., 2021; Takeuchi et al., 2021; Angin et al., 2021). Additionally, LCR had been associated with infectious diseases. A meta-analysis conducted by Lagunas-Rangel (2020) based on six studies revealed a potential link between decreased LCR and the severity of COVID-19. Ullah et al. (2020) reported that a lower LCR could serve as a predictive marker for in-hospital complications and mortality in COVID-19 patients. Given its potential, LCR could be valuable in identifying and anticipating the severity and fatality of COVID-19. In a recent study involving 1035 COVID-19 patients in 2023, the authors observed that LCR was a predictive marker for severe forms of COVID-19 upon emergency department admission, suggesting its utility in identifying high-risk patients (Abensur Vuillaume et al., 2023). These findings collectively highlighted LCR as an easily accessible and objective hematological biomarker for systemic inflammation. However, limited attention had been given to the prognostic relationship between LCR and sepsis patients in the ICU in published research. As far as we know, this was the initial investigation exploring the potential impact of LCR in predicting the clinical outcomes of sepsis in patients. Our data indicated that a decreased LCR level in sepsis patients was linked to an increased risk of 30-day mortality and the occurrence of AKI. These results implied that an early reduction in LCR could function as a prospective indicator of unfavorable outcomes in sepsis patients.

Additionally, this study conducted a subgroup analysis to further analyze the risk stratification in different patient groups. The subgroup analysis results showed that the predictive significance of LCR for 30-day mortality and AKI occurrence remained stable across male and female patients. Nonetheless, there was no noteworthy correlation observed between LCR and either 30-day mortality or AKI incidence among patients with hypertension and diabetes who were part of this study. This could be attributed to reverse causality, where individuals with these underlying conditions were more inclined to receive suitable treatment or embrace healthier lifestyle choices, potentially impacting the connection between LCR and the outcomes. Another important finding of our study was the observation that patients with lower LCR values tended to be older, and the association between LCR and 30-day mortality or AKI occurrence appeared to be more pronounced in this older patient group. It was imperative for healthcare providers to give heightened consideration to older patients due to their potentially higher prevalence of comorbidities. However, our study underscored the necessity of providing equal focus on younger patients as well, considering that they may still face a high mortality rate despite their relatively younger age.

The study presented in this article had a significant strength in demonstrating the correlation between a decrease in LCR and poor outcomes in sepsis patients, establishing LCR as an important independent risk factor. However, there were several limitations that require attention. Firstly, it was important to note that this research was a single-center study with a relatively small sample size. In order to validate the accuracy of our findings, conducting multicenter studies with larger sample sizes was necessary. Secondly, because of the retrospective nature of the study, selection bias and missing data cannot be entirely ruled out. Although we established strict and clear inclusion and exclusion criteria, as well as unified data collection methods, prospective studies are still needed to confirm the prognostic value of the LCR in real-time clinical decision-making. Thirdly, despite efforts made to address confounding variables through adjustments for multiple factors and subgroup analyses, there may still be undetermined factors affecting the prognosis, such as the use of antibiotics, fluid resuscitation, corticosteroids, and organ dysfunction, which were not taken into account due to lack of available data. We will consider the importance of these research variables in future studies to ensure comprehensive results. Fourthly, all participants were exclusively Chinese patients, warranting further investigation into the association between LCR and short-term outcomes within different populations. Moreover, this analysis focused solely on the prognostic value of baseline LCR, leaving uncertainty regarding the predictive value of LCR changes during follow-up. Therefore, the predictive power of the LCR change is also needed to be evaluated in future research.

## Conclusion

Our study findings highlighted the importance of LCR as a valuable indicator for predicting 30-day mortality and AKI occurrence in sepsis patients. Therefore, incorporating LCR measurement into the assessment of risk and prognosis for this patient population could prove beneficial. Furthermore, future research should concentrate on exploring whether interventions aimed at the LCR could improve clinical outcomes in these patients.

## Data Availability

The original contributions presented in the study are included in the article/Supplementary Material, further inquiries can be directed to the corresponding author.
