# Supplementary material for: The value of lymphocyte-to-C-reactive protein ratio for predicting clinical outcomes in patients with sepsis in intensive care unit: a retrospective single-center study
Source: Front Mol Biosci. 2024 Sep 13;11:1429372. doi: 10.3389/fmolb.2024.1429372 (PMC11427359; doi:10.3389/fmolb.2024.1429372)
Supplement: Supplementary file 1 [file Table1.docx]

**Supplementary material**

Supplementary Table 1: Baseline characteristics of the Q1-Q3 and Q4 groups

Supplementary Table 2: The diagnostic accuracy of various prediction factors for 30- day mortality and AKI

occurrence

Supplementary Table 3: Cox proportional hazards regression of the factors influencing 30-day mortality of the study population.

Supplementary Table 4: Binary logistic regression analysis of the factors influencing AKI occurrence of the study population.

Table S1. Baseline characteristics of the Q1-Q3 and Q4 groups

| **Variables** | **Overall** | **Q1-Q3 group** | **Q4 group** | **P-value** |
| --- | --- | --- | --- | --- |
| N | 1123 | 842 | 281 |  |
| Age, years | 75 (65-84) | 76 (66-84) | 74 (64-85) | 0.635 |
| Male, n (%) | 707 (63.0) | 541 (64.3) | 166 (59.1) | 0.120 |
| BMI, kg/m^2^ | 22.49 (20.08-25.21) | 22.49 (20.11-25.15) | 22.49 (19.99-25.26) | 0.640 |
| Smoking, n (%) | 229 (20.4) | 181 (21.5) | 48 (17.1) | 0.110 |
| **Comorbidities, n (%)** | | | | |
| Hypertension | 579 (51.3) | 442 (52.5) | 137 (48.8) | 0.277 |
| Diabetes | 309 (27.5) | 234 (27.8) | 75 (26.7) | 0.721 |
| Coronary artery disease | 116 (10.3) | 94 (11.2) | 22 (7.9) | 0.115 |
| COPD | 87 (7.7) | 58 (6.9) | 29 (10.3) | 0.062 |
| Cerebral infarction | 161 (14.3) | 121 (14.4) | 40 (14.2) | 0.955 |
| **Infection pathogens, n (%)** | | | | |
| Gram-positive bacteria | 136 (12.1) | 103 (12.2) | 33 (11.7) | 0.828 |
| Gram-negative bacteria | 335 (29.8) | 271 (32.2) | 64 (22.8) | 0.003 |
| Fungus | 77 (6.9) | 69 (8.2) | 8 (2.8) | 0.002 |
| Virus | 60 (5.3) | 49 (5.8) | 11 (3.9) | 0.219 |
| **Laboratory tests** | | | | |
| WBC *10^9^ /L | 11.4 (7.4-17.1) | 11.5 (7.4-17.4) | 11.0 (7.3-16.2) | 0.312 |
| Neu *10^9^ /L | 10.1 (6.3-15.5) | 10.4 (6.6-16.1) | 9.3 (6.0-14.4) | 0.006 |
| Lym *10^9^ /L | 0.6 (0.3-0.9) | 0.5 (0.3-0.7) | 0.9 (0.6-1.4) | <0.001 |
| Mon *10^9^ /L | 0.4 (0.2-0.7) | 0.4 (0.2-0.6) | 0.5 (0.2-0.8) | 0.003 |
| Hb, g/dL | 115 (97-130) | 112 (95-128) | 120 (105-138) | <0.001 |
| PLT *10^9^ /L | 149 (95-214) | 136 (86-201) | 189 (141-254) | <0.001 |
| CRP, mg/L | 104.2 (42.0-163.2) | 132.5 (88.1-186.6) | 12.3 (4.1-31.3) | <0.001 |
| LCR | 5.62 (2.56-16.35) | 3.91 (2.08-7.20) | 64.71 (29.01-197.56) | <0.001 |
| Tbil, μmol/L | 17.4 (10.9-28.2) | 18.4 (11.9-30.1) | 14.0 (8.4-23.2) | <0.001 |
| ALT, U/L | 32.0 (21.0-56.0) | 32.3 (21.2-58.3) | 31.0 (20.1-50.0) | 0.113 |
| AST, U/L | 38.1 (23.9-73.0) | 40 (25-81) | 32.0 (21.0-62.0) | 0.001 |
| Albumin, g/L | 28.2 (24.2-33.2) | 27.4 (23.7-32.1) | 31.6 (27.3-36.4) | <0.001 |
| Glucose, mmol/L | 8.2 (6.6-11.8) | 8.4 (6.6-12.1) | 7.9 (6.5-10.1) | 0.028 |
| Creatinine, μmol/L | 92.6 (63.7-153.1) | 110.0 (66.8-165.5) | 76.1 (56.5-122.1) | <0.001 |
| BUN, mmol/L | 8.89 (6.04-13.95) | 9.77 (6.37-14.98) | 7.11 (5.18-10.28) | <0.001 |
| Uric acid, μmol/L | 286.9 (192.3-411.7) | 293.2 (192.8-422.8) | 274.4 (189.2-384.7) | 0.053 |
| D-dimer, mg/L | 4.2 (2.1-8.4) | 4.8 (2.4-8.8) | 3.4 (1.6-7.5) | <0.001 |
| Potassium, mmol/L | 3.7 (3.3-4.2) | 3.7 (3.3-4.1) | 3.8 (3.3-4.2) | 0.111 |
| Lactate, mmol/L | 2.1 (1.4-3.6) | 2.1 (1.5-3.7) | 2.0 (1.3-3.4) | 0.011 |
| **Severity scoring** |  |  |  |  |
| APACHE II score | 25 (19-30) | 25 (20-30) | 25 (18-30) | 0.272 |
| SOFA score | 12 (10-14) | 12 (10-14) | 13 (10-15) | 0.006 |
| **Treatments** | | | | |
| CRRT, n (%) | 78 (6.9) | 66 (7.8) | 12 (4.3) | 0.042 |
| Vasoactive drug, n (%) | 748 (66.6) | 599 (71.1) | 149 (53.0) | <0.001 |
| Invasive ventilation, n (%) | 752 (67.0) | 553 (65.7) | 199 (70.8) | 0.113 |
| **Endpoints** | | | | |
| 30-day mortality, n (%) | 316 (28.1) | 270 (32.1) | 46 (16.4) | <0.001 |
| AKI, n (%) | 512 (45.6) | 423 (50.2) | 89 (31.7) | <0.001 |
| Length of ICU stay, days | 6 (3-12) | 6 (3-12) | 6 (3-12) | 0.194 |
| Length of hospital stay, days | 16 (11-25) | 16 (11-25) | 17 (9-27) | 0.720 |
| 60-day mortality, n (%) | 375 (33.4) | 317 (37.6) | 58 (20.6) | <0.001 |
| ICU mortality, n (%) | 358 (31.9) | 303 (36.0) | 55 (19.6) | <0.001 |
| Hospital mortality, n (%) | 379 (33.7) | 320 (38.0) | 59 (21.0) | <0.001 |

Abbreviations: LCR, Lymphocyte-to-C-Reactive Protein ratio; BMI, body mass index; COPD, chronic obstructive pulmonary disease; WBC, white blood cell count; Neu, neutrophil; Lym, lymphocyte; Mon, monocyte; Hb, hemoglobin; PLT, platelet; CRP, C-reactive protein; Tbil, total bilirubin; ALT, alanine transaminase; AST, aspartate aminotransferase; BUN, blood urea nitroge; APACHE II, Acute Physiology and Chronic Health Evaluation II; SOFA, Sequential Organ Failure Assessment; CRRT, continuous renal replacement therapy; AKI, Acute kidney injury; ICU, Intensive Care Unit.

Table S2. The diagnostic accuracy of various prediction factors for 30-day mortality and AKI

occurrence

| Variables | 30-day mortality | | | | | AKI occurrence | | | | |
| --- | --- | --- | --- | --- | --- | --- | --- | --- | --- | --- |
|  | AUC (95%CI) | Cut-off value | Sensitivity | Specificity | P-value | AUC (95%CI) | Cut-off value | Sensitivity | Specificity | P-value |
| LCR | 0.620 (0.585-0.656) | 6.09 | 0.649 | 0.543 | <0.001 | 0.625 (0.592-0.657) | 4.49 | 0.541 | 0.666 | <0.001 |
| WBC | 0.543 (0.506-0.580) | 12.6 | 0.506 | 0.582 | 0.024 | 0.583 (0.549-0.617) | 12.6 | 0.529 | 0.630 | <0.001 |
| Neu | 0.553 (0.516-0.590) | 8.2 | 0.690 | 0.403 | 0.005 | 0.590 (0.556-0.624) | 11.2 | 0.543 | 0.628 | <0.001 |
| Lym | 0.614 (0.579-0.649) | 0.8 | 0.387 | 0.794 | <0.001 | 0.556 (0.522-0.590) | 0.7 | 0.658 | 0.455 | 0.001 |
| CRP | 0.552 (0.516-0.589) | 30.9 | 0.883 | 0.238 | 0.006 | 0.620 (0.588-0.653) | 121.5 | 0.539 | 0.659 | <0.001 |
| Albumin | 0.560 (0.523-0.598) | 27.2 | 0.611 | 0.525 | 0.002 | 0.562 (0.528-0.596) | 27.9 | 0.547 | 0.590 | <0.001 |
| APACHE II score | 0.612 (0.576-0.648) | 27 | 0.504 | 0.644 | <0.001 | 0.607 (0.574-0.640) | 28 | 0.471 | 0.706 | <0.001 |
| SOFA score | 0.608 (0.572-0.644) | 13 | 0.491 | 0.638 | <0.001 | 0.605 (0.572-0.638) | 13 | 0.519 | 0.650 | <0.001 |

Abbreviations: LCR, Lymphocyte-to-C-Reactive Protein ratio; WBC, white blood cell count; Neu, neutrophil; Lym, lymphocyte; CRP, C-reactive protein; APACHE II, Acute Physiology and Chronic Health Evaluation II; SOFA, Sequential Organ Failure Assessment; AKI, Acute kidney injury.

Table S3. Cox proportional hazards regression of the factors influencing 30-day mortality of the study population.

| **Variables** | **HR** | **95% CI** | **P-value** |
| --- | --- | --- | --- |
| LCR | 0.351 | 0.131-0.938 | 0.037 |
| Age | 1.017 | 1.009-1.026 | <0.001 |
| Male | 1.084 | 0.861-1.365 | 0.492 |
| BMI | 0.964 | 0.939-0.991 | 0.008 |
| Hypertension | 1.140 | 0.913-1.424 | 0.248 |
| Diabetes | 1.056 | 0.826-1.350 | 0.662 |
| WBC | 1.007 | 0.995-1.018 | 0.254 |
| Neu | 1.016 | 1.002-1.030 | 0.023 |
| Hb | 0.990 | 0.985-0.994 | <0.001 |
| Lactate | 1.095 | 1.066-1.125 | <0.001 |
| APACHE II score | 1.047 | 1.034-1.060 | <0.001 |
| SOFA score | 0.982 | 0.951-1.014 | 0.260 |
| Invasive ventilation | 3.555 | 2.512-5.030 | <0.001 |

Abbreviations: LCR, Lymphocyte-to-C-Reactive Protein ratio; BMI, body mass index; WBC, white blood cell count; Neu, neutrophil; Hb, hemoglobin; APACHE II, Acute Physiology and Chronic Health Evaluation II; SOFA, Sequential Organ Failure Assessment.

Table S4. Binary logistic regression analysis of the factors influencing AKI occurrence of the study population.

| **Variables** | **OR** | **95% CI** | **P-value** |
| --- | --- | --- | --- |
| LCR | 0.484 | 0.274-0.856 | 0.013 |
| Age | 1.004 | 0.996-1.012 | 0.316 |
| Male | 0.921 | 0.722-1.175 | 0.508 |
| BMI | 1.013 | 0.985-1.042 | 0.352 |
| Hypertension | 1.224 | 0.967-1.548 | 0.093 |
| Diabetes | 1.488 | 1.144-1.935 | 0.003 |
| WBC | 1.044 | 1.027-1.061 | <0.001 |
| Neu | 1.054 | 1.036-1.073 | <0.001 |
| Hb | 0.992 | 0.987-0.997 | 0.001 |
| Lactate | 1.310 | 1.232-1.393 | <0.001 |
| APACHE II score | 1.050 | 1.034-1.066 | <0.001 |
| SOFA score | 0.970 | 0.938-1.003 | 0.076 |
| Invasive ventilation | 0.939 | 0.732-1.206 | 0.624 |

Abbreviations: LCR, Lymphocyte-to-C-Reactive Protein ratio; BMI, body mass index; WBC, white blood cell count; Neu, neutrophil; Hb, hemoglobin; APACHE II, Acute Physiology and Chronic Health Evaluation II; SOFA, Sequential Organ Failure Assessment; AKI, Acute kidney injury.
